# Empirical investigation on how wellbeing-related infrastructure shapes economic growth: Evidence from the European Union regions

**DOI:** 10.1371/journal.pone.0283277

**Published:** 2023-04-19

**Authors:** Larissa M. Batrancea, Anca Nichita, Mehmet Ali Balcı, Ömer Akgüller

**Affiliations:** 1 Department of Business, Babes-Bolyai University, Cluj-Napoca, Romania; 2 Faculty of Economics, “1 Decembrie 1918” University of Alba Iulia, Alba Iulia, Romania; 3 Department of Mathematics, Muğla Sıtkı Koçman University, Muğla, Turkey; Università Cattolica del Sacro Cuore Sede di Piacenza e Cremona Facoltà di Economia: Universita Cattolica del Sacro Cuore Facolta di Economia e Giurisprudenza, ITALY

## Abstract

One of the most important policies of the European Union is regional development, which comprises measures of enhancing economic growth and citizens’ living standards via strategic investment. Considering that economic growth and wellbeing are intertwined from the perspective of EU policies, this study examines the relationship between wellbeing-related infrastructure and economic growth in 212 NUTS 2 regional subdivisions across the members of Eu-28 during the period 2001–2020. We therefore analyzed data from 151 Western Europe regions and 61 Central and Eastern Europe regions by means of a panel data analysis with the first-difference generalized method of moments estimator. Our main interest was to determine the degree to which Western Europe regions responded to predictors as compared to Central and Eastern Europe regions. According to the empirical results, the predictors with the strongest influence for Western Europe regions were disposable household income, inter-regional mobility, housing indicator, labor force and participation. For Central and Eastern Europe regions, the largest impact was triggered by the housing indicator, internet broadband access and air pollution. In addition, we determined a relational weighted multiplex between all variables of interest by using dynamic time warping and we introduced topological measures in a multilayer multiplex model for both regional subsamples.

## 1. Introduction

The phenomenon of economic growth can be regarded as an important tool “for reducing poverty and improving the quality of life” of people around the world on the ground that it can “generate virtuous circles of prosperity and opportunity”. When people seize job opportunities available on the market and take challenges to improve their lives, they become incentivized to support the education and training of family members. This in turn stimulates entrepreneurial initiatives and the growth of products and services catered on the market [1].

Economic growth (i.e., economic progress or performance) captures an increase in the overall capacity of a country to produce goods and services over a period of time. The economic literature pinpoints that the main macroeconomic indicators used in assessing economic growth for national economies and regions [2–9] are gross domestic product and gross value added.

The well-established benchmark indicator *gross domestic product* (GDP) was introduced to the literature in 1937 by Simon Kuznets [10], an economist at the National Bureau of Economic Research and Nobel Prize winner “for his empirically founded interpretation of economic growth which has led to new and deepened insight into the economic and social structure and process of development”. GDP measures the monetary value of all goods and services yearly produced for final consumption within an economy. The concept of *gross value added* (GVAD) is strictly connected to the GDP in that it captures the value of goods and services of an economy without considering the intermediate consumption (i.e., the value of goods and services needed to yield the final goods and services is therefore subtracted).

The economic literature of recent decades reports a rich stream of studies tackling the matter of economic growth and its main determinants [11]. In their comprehensive bibliometric analysis, Doré and Teixeira [12] divided economic growth determinants into the following categories: human capital; labor and demographics; technology, innovation and structural change; macroeconomic conditions; foreign direct investment and international trade; geography and natural resources; institutional contexts. Hence, with the passage of time, the literature has acknowledged the major influence of certain determinants such as demography [13], education [14], European integration [15], financial inclusion, labor and trade openness [16], healthcare expenditure [9, 17], income inequality [18] and inflation [19], to mention but a few.

For that matter, studies on the phenomenon of economic growth are far from being exhaustive, there is still room for a host of new scientific inquiries and answers that can be added to the worldwide scientific conversation. In this sense, considering the complexity of the phenomenon, the stream of studies dedicated to economic growth has been constantly growing, especially when it comes to empirical investigations.

The abundance of studies has elicited a variety of methods to analyze economic growth, ranging from the conventional qualitative and quantitative literature reviews, systematic literature reviews and bibliometric analyses [12, 18–21] to more technical insights via Bayesian Averaging of Classical Estimates [22], Bayesian panel data analysis [23], quantile-on-quantile regression analysis [9], panel data analysis [3, 16], pooled cross-country time-series data [24], as a case in point.

Researchers have also manifested a particular interest in conducting regional analyses on economic growth and highlighting specific insights on economic progress from different regions across the globe. Consequently, scientific literature counts multiple investigations focused on: Central and Eastern Europe countries [15, 25, 26]; old and new member states of the European Union [16, 27]; members of the Organisation for Economic Co-operation and Development (OECD) [24], Latin American economies [26, 28]; MENA economies [29]; various emerging and developed economies [30–32].

In direct connection with regional studies and regional policy of the European Union (EU), a new stream of research has emerged by considering the determinants of economic growth across development regions of the EU [33–37]. Regional policy-wise, some of the relevant determinants of economic growth across the EU are entrepreneurial culture [38], human capital [39, 40], social capital [41], regional convergence [42] or waste generation [43]. Still, in our view, empirical studies on development regions in the European Union are rather scarce and the literature would benefit a great deal from similar investigations, considering the structural changes that the Union has witnessed in the last two decades.

Within the framework of EU policies, the regional development policy is one of its most important policies because it comprises measures of enhancing economic growth and citizens’ living standards via strategic investment. According to the EU, the regional development policy targets five areas:

- Investment in people via creating opportunities for education, employment and social inclusion;
- Investment in small and medium businesses;
- Investment in research and innovation while increasing related-employment opportunities;
- Investment in environmental protection;
- Investment in transportation and energy production to counteract the negative externalities of climate change, favoring innovative transport infrastructure and renewable energy solutions.

Designed on the solidarity principle, the regional development policy is mainly based on financial solidarity, that is the redistribution of a part of the EU budget financed by member states to less prosperous regions and social groups. In other words, the abovementioned targeted investments aim to mitigate economic and social disparities among regions in the medium and long term.

What are the development regions in question? To keep track of economic and social contexts within member states under a unitary system, the European Union developed in 1988 a standard division called the Nomenclature of Territorial Units for Statistics (NUTS) (see Table 1). Therefore, regional analyses are carried out at three territorial levels and aim to identify: a) regional problems through comparative analyses of NUTS level 1 regions; b) regional problems of through comparative analyses of NUTS level 2 regions; c) local problems through comparative analyses of NUTS level 3 regions.

**Table 1:** NUTS classification for development regions.

Level	Minimum population number	Maximum population number
NUTS 1	3 million	7 million
NUTS 2	800,000	3 million
NUTS 3	150,000	800,000

*Source*: Regulation no. 1059 on establishing a common classification of territorial-statistical units [44].

Out of the three types of NUTS regions, the NUTS 2 regional subdivision is mostly used in connection with analyzing the regional absorption capacity of EU funds [45, 46] and identifying regional differences.

In relation to the EU policy framework, according to a concluding summary on the Economy of Wellbeing [47], the EU Council [48, 49] stresses that “people’s wellbeing is a principal aim of the European Union. […] At the same time, sustainable and inclusive economic growth and resilience function as enablers for the wellbeing of people, societies and the planet”. In this context, the EU Council also advocates for a balance between economic growth and social progress.

Starting from the importance of regional subdivisions within the European Union and citizens’ wellbeing degree, we focused on examining the impact of *wellbeing-related infrastructure* on the phenomenon of economic growth in 212 NUTS 2 regional subdivisions across the members of EU-28. We were mainly interested in determining the degree to which Western Europe (WE) regions responded to predictors as compared to Central and Eastern Europe (CEE) regions.

From the perspective of our study, the newly developed category of wellbeing-related infrastructure refers to the facilities (air quality, housing indicator, internet broadband access) and the capacity of accessing such facilities (income, mobility of human resources, labor force, engagement in education and training), which can shape the level of economic growth. For that matter, according to the EU, citizens’ wellbeing and economic growth are “interdependent and mutually reinforcing”. We deem that economies across EU regions are prone to economic performance when citizens have more disposable income at hand to acquire products and services, can move freely between regions in search for job opportunities, benefit from high-speed internet access (a *sine-qua-non* facility for nowadays digitalized world), live in larger housing facilities, participate on the labor market (provided they fit national working-age requirements) and thoroughly use education and training possibilities.

Hence, the set of independent variables that we considered comprises the following: air pollution (AP); housing indicator (HI); internet broadband access (IBA); disposable household income (DHI); inter-regional mobility (IRM); labor force and participation (LFP); labor utilization rate (LU); rate of early leavers from education and training (ELET). The proxies for economic growth were regional gross domestic product (GDP) and regional gross value added (GVAD). The period of analysis considered was 2001–2020 and the variables of interest were retrieved from the OECD regional database [50], which provided regional yearly observations across the entire time span.

To the best of our knowledge, this is the first study documenting the impact of wellbeing-related infrastructure on economic growth with a particular interest on potential differences between WE regional subdivisions and CEE regional subdivisions. We therefore fill the gap in the economic growth literature and provide compelling insights on measures and policies that could boost economic growth for WE countries and CEE countries and, at the same time, that could mitigate disparities among EU development regions.

We ran two econometric models for each of the regional subsamples (Western Europe, Central and Eastern Europe) to identify potential differences between the intensity of the predictors. According to our results, the chosen predictors played a significant role in shaping the evolution of economic growth during the two decades. Specifically, the independent variables that had the most notable influence in the case of WE development regions were disposable household income, inter-regional mobility, housing indicator, labor force and participation. In the case of CEE development regions, the largest impact was registered for internet broadband access, air pollution and housing indicator. With a few exceptions, all predictors had a relevant impact for both subsamples. The main differences stemmed from the strength of the influence and the sign of the relationships between economic growth and designated predictors.

We also investigated differences between WE and CEE regions by means of a multiplex network analysis and related metrics. Regarding multiplex correlations, when it comes to the WE regions, air pollution was highly correlated with labor utilization rate. In the case of CEE regions, high correlations were registered for the variable pairs air pollution – labor force and participation, as well as for internet broadband access – disposable household income. Both types of regions reported nodes that were truly multiplex: the CEE subsample registered such hubs in regions from Poland, while the WE subsample displayed hubs in France, Germany, the UK and Nordic states.

The remainder of the paper has the following structure. The *Literature Review* section highlights relevant studies tackling the factors that drive economic growth across EU regional subdivisions. The *Method* section emphasized the advantages of the chosen analytical approach, regional sample, period of analysis, variables of interest and research hypotheses, proposed econometric models. The *Results* section reports on the estimated outcomes. The final section briefly presents the main results of the study, lists policy implications and identifies future research directions.

## 2. Literature review

As previously stated, the literature acknowledges studies that focused on determinants of economic growth at regional level within the EU. Cartone, Postiglione and Hewings [51] investigated differences in the determinants of economic growth for 187 regions across 12 European countries from 1981 to 2009. Empirical results elicited that such differences stemmed from convergence rates, investments, population growth, human capital and spillovers. With data from 268 EU regions for the period 2000−2014, Liu [52] focused on determinants of regional economic growth. His research showed that regional communication was positively linked to economic growth, but population and *education level* were negatively connected to the dependent variable.

Starting from the pioneering research on social capital introduced by Putnam in 1993, the authors Schneider, Plümper and Baumann [41] examined the link between economic progress, various economic indicators and aspects of political culture (i.e., communication, trust, prevalence of traditional education attitudes in a region) for 56 NUTS 2 regions in the EU for the period 1980–1996. Empirical results showed that the strongest drivers of economic growth were economic variables such as regional capital formation, share of government consumption, economic openness. Yet, the prevalence of these economic indicators is expected considering the analyzed time frame. Along the years, other important drivers and disrupters of economic growth have emerged: the set of drivers includes catch-up potential, demographics (“projected growth in labor force”), investment size and productivity; the set of disrupters includes automation, climate change, digitization, political populism and protectionism [53].

Based on historical European regions that were adapted to fit the current NUTS 2 classification system, Diebolt and Hippe [40] conducted a compelling investigation on the impact of *human capital* on innovation and economic growth for the period 1850–2010 and 256 regional subdivisions. By means of standard OLS regression modelling, they concluded that nowadays level of economic performance across the EU regions was considerably determined by the human capital formation in the long run. Consequently, the authors advocated for the necessity of strategic investments in human capital to keep augmenting economic growth, while taking into account the characteristics of each regional subdivision.

Agasisti and Bertoletti [54] examined the impact of regional higher education systems on economic growth using data from 284 NUTS 2 regions spanning 2000−2017. Results indicated that regional economic growth was positively impacted by a growing number of universities.

Based on data provided by 280 European regions during 2006−2015, Bianchi, del Valle and Tapia [55] studied the effects of regional economic structures on the socioeconomic determinants of material productivity. Authors noted that higher affluence generated higher material productivity gains in material-intensive regions as compared to service-oriented regions. In addition, urban agglomeration degree was the most relevant driver of material productivity, which had a bigger impact on densely populated regions.

Goschin [56] examined the spatial correlation between *real wage earnings* and regional economic performance in Romania with the help of panel data modeling. Results showed that development level proxied by GDP per capita influenced regional wage determination in the long run. Using data from seven EU countries during 1990−2005, Benos, Karagiannis and Karkalakos [57] investigated the influence of proximity on regional growth. According to their findings, spillovers played a fundamental role in regional growth, irrespective of how proximity was measured. Namely, authors concluded that regions in proximity of dynamic entities would grow faster. Their results emphasized the importance of implementing policies that favor human capital accumulation, considering regional synergies.

Ramajo et al. [58] examined convergence speed for 163 EU regions during the period 1981–1996. Empirical results showed that regions within cohesion-fund countries (e.g., Greece, Ireland, Portugal, Spain) converged at a different speed than other regions. Völlmecke, Jindra and Marek [59] studied income convergence for 269 EU regions between 2003 and 2010. Results highlighted a weak income convergence across regions, with Central and East European regions being the outliers. By using data from 18 EU countries for the time span 1997−2017, Yu, Fang and Dong [60] focused on the convergence process regarding the generation of thermal electricity and its influence on economic growth. Mewes and Broekel [61] studied how technological complexity of activities influenced regional economic growth on a sample of 159 European NUTS 2 regions for the period 2000−2014. According to their results, if a country’s level of technological complexity increased by 10%, its economic growth measured via GDP per capita would improve by 0.45%, hence supporting the implementation of smart specialization strategies [62].

With NUTS 3 data for the budgetary periods 1994–1999 and 2000–2006, Becker, Egger and von Ehrlich [63] examined the impact of regional funds and transfers on economic growth. Results indicated that fund reallocation across regions increased economic growth and generated convergence to a greater extent. Kilroy and Ganau [64] retrieved data from 1,321 NUTS 3 regions for the period 2003–2017 to analyze the impact of industrial structure, innovation and inflow of foreign direct investment on economic progress. According to their results, low-income regions registered a strong effect of foreign direct investment. At the same time, variables such as construction and innovation played an important role for the economic growth of non-rural regions.

Scientific literature also reports multiple studies investigating the state of economic development, which grasps economic growth and any improvement in the quality of life. According to a report issued by the UK Department for International Development [1], economic growth and economic development are organically linked since a significant economic growth “advances human development, which, in turn, promotes economic growth”. For that matter, the main policy goal of a country is to stimulate economic output as a necessary base for economic and social development.

In this context, Fila and Kucera [65] assessed the connection between innovation and economic development in Slovakian regions. Biagi, Brandano and Ortega-Argiles [66] investigated the impact of tourism-related strategies on EU regional development and reported that tourism was considered a priority for almost half of their sample, which included regions of different development levels. With data from lagging-behind European regions during the time frame 2002−2008, De Noni and Belussi [67] studied the impact of local innovative organizations on the development of regional inventor networks. Their results indicated that collaborative, highly innovative regions had a positive influence on innovation performance.

According to the regional development policy of the European Union, the overall development level of a country is organically linked to the economic growth and development of its regional subdivisions. In this context, a state could grow its long-term production of goods and services when disparities among regional subdivisions are mitigated via adequate strategies and sensible investment in infrastructure projects [68, 69]. In our view, even though a country (or an association of countries) might register a growth at national (regional) level, the existence of conspicuous differences between its subdivisions may hinder economic progress in the long run. From the perspective of this study, we deem that a high level of the wellbeing-related infrastructure can mitigate such differences and steer regional economic growth on an upward trend.

The importance of *environment*, *housing*, *income*, *jobs* and their link to citizens’ wellbeing captured by material living conditions and quality of life is emphasized through the composite indicator *Better Life Index* launched in 2011 by the Organisation for Economic Co-operation and Development [70]. This indicator captures stances of wellbeing in relation to the following dimensions: housing; income; jobs; community; education; environment; civic engagement; health; life satisfaction; safety; work-life balance.

According to the OECD composite indicator, the housing dimension comprises elements such as the average number of rooms per person, the percentage of dwellings with basic facilities (water, sewage supply) and housing expenditure (e.g., utilities, rent, amenities, repairs and home modifications). The dimension of income refers to household net wealth (aggregated wealth comprising financial and non-financial assets, net liabilities) and household net adjusted disposable income (money earned annually, to be spent on goods and services, once taxed are deducted). The dimension related to environment captures indicators such as air pollution and water quality. Last but not least, the dimension regarding jobs includes indicators such as job security, personal earnings, long-term unemployment rate and employment rate.

Hence, considering the input from the scientific literature and international organizations, wellbeing-related infrastructure is a category that should be considered when analyzing economic growth across regional subdivisions. The following paragraphs provide details on the variables of interest included in the proposed category and the chosen proxies for the phenomenon of economic growth, which will be empirically analyzed at regional level.

## 3. Method

### 3.1. Study sample, variables of interest, period of analysis

#### 3.1.1 Sample of regional subdivisions

Our study focused on the regional data across the members of EU-28, divided into two subsamples: *Western Europe (WE)* – Austria, Belgium, Cyprus, Denmark, Finland, France, Germany, Greece, Ireland, Italy, Luxembourg, Malta, the Netherlands, Portugal, Spain, Sweden, United Kingdom; *Central and Eastern Europe (CEE)* – Bulgaria, Croatia, Czech Republic, Estonia, Hungary, Latvia, Lithuania, Poland, Romania, Slovenia, Slovakia.

A division of the 28 states into the two subsamples is sensible and standard when it comes to European Union statistics. Moreover, this division is based on the common geopolitical background of these countries. Namely, WE states are established democracies, developed nations, experienced market economies, founding and/or early members of the Union.

Located in the Baltic area, Central and Eastern Europe, CEE states are former communist countries belonging to the Soviet bloc, which joined the EU at later stages. There are several reasons for which CEE countries are researched less in the extant literature. Hence, the first reason is that CEE economies have been closed for long periods because of their political regimes. The second reason resides in their centralized economies, which were grounded on the socialist production model that regulated business prospects during the communist regime. The third reason is that state policies have been secluded from the market economy. Moreover, the lack of competition mitigated survival challenges, which triggered a low motivation for economic growth. Central governments established the nature of funded business projects. Therefore, people participated less in capital formation and investment choices. Capital was a state instrument, there was less or no room for free flow of foreign direct investment and the life cycle of the product was controlled by the state.

We focused on a total of 212 regional subdivisions according to the NUTS 2 classification, which included 151 WE regions and 61 CEE regions corresponding the EU-28 states (see the S1 Appendix).

We conducted econometric analyses and complex network analysis for the subsample of Western Europe regions and the subsample of Central and Eastern Europe regions.

#### 3.1.2 Variables of interest

Table 2 displays the variable symbols and corresponding descriptions. All variables were retrieved from the regional database compiled by the Organisation for Economic Co-operation and Development.

**Table 2:** Variable symbols and descriptions.

Variable name	Symbol	Description
**1) Dependent variables**		
**Regional gross domestic product**	**GDP**	Monetary value of all goods and services yearly produced for final consumption within a region
**Regional gross value added**	**GVAD**	Monetary value of goods and services yearly produced within a region, less the intermediate consumption
**2) Independent variables**		
**Air pollution**	**AP**	Average level in μg/m³ experienced by the population of a region
**Housing indicator**	**HI**	Average number of rooms per household inhabitant (rooms per capita)
**Internet broadband access**	**IBA**	Share of households with internet broadband access (percent in total households)
**Disposable household income**	**DHI**	Income available to households after taxpaying (included in the category “Regional income per capita”)
**Inter-regional mobility**	**IRM**	Mobility of new residents between regions from the same country
**Labor force and participation**	**LFP**	Working age population between 15–64 years old
**Labor utilization rate**	**LU**	Percentage of total employment in the regional population
**Rate of early leavers from education and training**	**ELET**	Percentage in the total population aged 18−24

*Source*: https://stats.oecd.org/ [50].

The phenomenon of economic growth was proxied by two relevant variables: regional gross domestic product and regional gross value added. The category of wellbeing-related infrastructure was captured by the eight predictors presented in the table above, linked to environment, housing, internet access, income, mobility of human resources, labor force, engagement in education and training.

#### 3.1.3 Period of analysis

Considering the nature of the economic phenomenon, we aimed at examining the potential long-term link between wellbeing-related infrastructure and economic growth across the 212 regional subdivisions. Therefore, we focused on the period spanning 2001–2020 since the OECD regional database provided observations for all our variables of interest and all regional subdivisions from Western Europe and Central and Eastern Europe, thus providing balanced panel data.

### 3.2. Econometric modelling

We conducted a panel data modeling using the first-difference general method of moments (GMM) approach with cross-section fixed effects since it produces robust outcomes while controlling for heteroscedasticity and endogeneity problems [71, 72].

The general form of the econometric model is the following:

$$\begin{array}{l} In\left(Y_{it}\right)=a_{0}+a_{1}X_{1it}+a_{2}In\left(X_{2it}\right)+a_{3}X_{3it}+a_{4}X_{4}{}_{it}+a_{5}In\left(X_{5it}\right)+a_{6}X_{6it}+a_{7}X_{7it}\\ +a_{8}In\left(X_{8it}\right)+\delta_{i}+\theta_{t}+\varepsilon_{it} \end{array}$$

where,

- *a_0_* represents the intercept;
- *a_i_* represents the coefficient of the independent variable, taking values from 1 to 8;
- *X* represents the independent variable;
- *i* indicates the region, taking values from 1 to 61 for Central and Eastern Europe regions and from 1 to 151 for Western Europe regions;
- *t* refers to the time period (i.e., 2001–2020);
- *δ_i_* captures the fixed effects that control for time-invariant region-specific factors;
- *θ_t_* captures the fixed effects that control for common shocks;
- *ε_it_* is the error term.

Our empirical research tested the following research hypotheses for both Western Europe regional subdivisions and Central and Eastern Europe regional subdivisions:

Hypothesis 1: There is a statistically significant relationship between regional gross domestic product and the predictors air pollution, housing indicator, internet broadband access, disposable household income, inter-regional mobility, labor force and participation, labor utilization rate and rate of early leavers from education and training.

Hypothesis 2: There is a statistically significant relationship between regional gross value added and the predictors air pollution, housing indicator, internet broadband access, disposable household income, inter-regional mobility, labor force and participation, labor utilization rate and rate of early leavers from education and training.

Bearing in mind that the European Union has evolved over time until it reached the EU-28 membership structure and that member states joined the union in different enlargement waves (with all CEE countries joining after 2004), we expect to identify certain differences in the strength of predictors between Western Europe regions and Central and Eastern Europe regions. Such differences would be sensible because of disparities in terms of economic growth, economic development, overall experience on the single market, number of catching-up regions and lagging regions [73].

### 3.3. Complex network analysis

From the standpoint of systems theory, the European NUTS classification is a complex system. According to the theory, complex systems often consist of multiple subsystems and link layers, which develop locally and globally under the influence of internal and external dynamics.

Generally, social networks interactions can be viewed as a collection of interactions at independent levels, representing different scenarios belonging to different identities. Although individual behavior is different at each level, behaviors are limited by different levels. Therefore, when analyzing relationships from the same social network, the relationship with people from one’s own layer and the relationship with people from different layers should be considered. For example, the question of infodemic that has recently emerged in social networks can be analyzed by examining complex interactions at different levels [74].

Multilayer networks have been successfully used in studies on disease spread [75, 76] or the evolution of cooperation in the presence of social dilemmas [77]. The most difficult area of complex systems theory is to analyze and predict system dynamics with many scales and components [74]. To overcome this difficulty, it is necessary to generalize the classical network theory. Nowadays, huge amounts of data are produced every second and making meaningful conclusions based on such data generates multiple benefits in almost every field.

Especially in social sciences, there is a need to switch from simple networks to complex networks [78] since empirical data and randomness have features that cannot be revealed with simple networks. Complex networks are effective when empirically analyzing real-world problems and relevant studies have been conducted on the theory and application of complex networks to different fields, including economics [79–88].

Macroeconomic markets and regions can be thought of as networks [89–91] and anything from an individual to a particular economic area can be deemed a node in a network. In this context, the possible types of connections between nodes are represented by network links.

The utility of networks resides in their capacity to explain the interconnectedness degree of the modern world via defining elements (i.e., multiple nodes and/or links) [92, 93]. Instead of focusing on individual parts of a system, network models provide an overall perspective, which is crucial when wanting to identify the system elements that need attention before designing a particular public policy. Hence, network analysis can be used to scrutinize the economic behavior of countries or regions and the attractiveness of certain industries. Network analysis can also serve to examine value chains, economic activities and their dependence on technology and external resources [94, 95].

Since network methodologies may be implemented to different settings, network science is fundamental to public policy. For that matter, due to their interconnected nature, socioeconomic systems pose a challenge for policymakers and regulators. In this context, an accurate mapping of the complexity of technological, economic and social ties can assist policymakers and regulators with designing and enacting policies [96, 97].

The specific features of time series corresponding to economic variables may serve to construct networks replicating interactions between countries and/or regions. The topologies of weighted networks can be generated combinatorically and graphically via distance functions computed on time series of multiple variables. Hence, in our study, besides the conventional econometric approach, we also used multiplex multilayer network topologies to illustrate multivariate interactions.

## 4. Results

### 4.1. Analysis of central tendency and variation

We conducted analyses by means of the statistical software EViews version 10.

Table 3 displays the descriptive statistics (mean, median, standard deviation, skewness, kurtosis) computed for the period 2001–2020 corresponding to the 151 Western Europe regions.

**Table 3:** Descriptive statistics for Western Europe regions.

Variables	GDP	GVAD	ELET	DHI	IBA	LU	IRM	AP	HI	LFP
Mean	106,820.5	98,351.75	15.024	18,443.48	72.605	44.879	38104.84	12.444	1.702	1,715,467
Median	58,906.8	53,601.6	12.5	18,084	78	44.7	22852	12.13	1.8	1,020,000
Maximum	884,510	793,461	56.5	37,624	100	75.8	282,739	32.073	2.66	1,186,4000
Minimum	1,023.77	1,176.92	3.5	3,132	3.2	16	268	3.599	1.1	17,000
Std. dev.	135,578	125,913.6	8.355	4,249.875	20.228	7.639	43,500.82	4.283	0.317	1,858,186
Skewness	2.834	2.811	1.679	0.358	−1.101	0.255	2.031	0.888	−0.087	2.108
Kurtosis	12.726	12.383	6.156	3.297	3.629	4.309	7.416	4.948	2.53	8.924
Jarque-Bera test	15,350.36***	12,205.02***	1,391.252***	66.607***	386.365***	199.704***	2,847.173***	601.673***	18.643***	5,266.670***
WE countries	17	17	17	17	17	17	17	17	17	17
Regions	151	151	151	151	151	151	151	151	151	151
Obs.	2,907	2,448	1,572	2,659	1,767	2,428	1,898	2,076	1,782	2,391

*Source*: Authors’ calculus.

*Note*: The symbol *** indicates significance at the 1% level.

As can be noticed from Table 3, according to the standard deviation values, the variables LFP, GDP and GVAD registered the largest volatility, while the variable HI registered the smallest volatility. According to the skewness values, the variables GDP, GVAD, ELET, DHI, LU, IRM, AP and LFP were skewed to the right, while the variables IBA and HI were skewed to the left. In terms of the kurtosis values, since the variables GDP, GVAD, ELET, DHI, LU, IRM, AP and LFP were above the threshold of 3, it means that their distributions were leptokurtic. At the same time, HI had a platykurtic distribution because its kurtosis value was below 3. To investigate the normal distribution of empirical data, we used the standard Jarque-Bera test. Under the null hypothesis of this test, data are normally distributed when the corresponding probability exceeds the chosen significance level. If the null hypothesis is rejected, it indicates that data are non-normally distributed. In the case of the Western Europe regions, the Jarque-Bera test showed that all eight predictors and two proxies for economic growth were non-normally distributed at the 1% level.

In the case of Central and Eastern Europe regions (Table 4), with respect to the standard deviations the variables LFP, GDP and GVAD registered the largest volatility, while the predictor HI registered the smallest volatility. According to the values of skewness, the variables GDP, GVAD, ELET, DHI, LU, IRM, AP, HI and LFP were skewed to the right, while the variable IBA was skewed to the left. Since the kurtosis values for variables GDP, GVAD, ELET, DHI, IBA, LU, IRM, AP and LFP were above the threshold of 3, the distributions of these variables were leptokurtic. Again, the variable HI had a platykurtic distribution. The Jarque-Bera test showed that all variables of interest were non-normally distributed at the 1% level.

**Table 4:** Descriptive statistics for Central and Eastern Europe regions.

Indicators	GDP	GVAD	ELET	DHI	IBA	LU	IRM	AP	HI	LFP
Mean	40,840.35	37,652.88	7.963	10,315.8	64.303	44.501	11,605.07	19.446	1.225	1,158,505
Median	33,246.3	31,550.9	7	10,044	71	43.3	7,572	18.879	1.2	964,300
Maximum	213,564	188,905	22.5	25,122	95	90.8	76,595	37.66	1.8	3,339,000
Minimum	8,564.4	7,540.06	1.6	2,972	0	27.2	1,114	5.73	0.87	432,000
Std. dev.	27,155.38	24935.47	4.038	3,865.381	21.337	10.043	12,460.67	4.843	0.203	563,880.7
Skewness	2.156	2.092	0.796	0.665	−1.147	1.741	2.661	0.516	0.466	1.298
Kurtosis	9.352	8.92	3.051	3.643	3.569	7.109	10.898	3.948	2.279	4.586
Jarque-Bera test	2,752.887***	2,067.440***	43.182***	92.278***	144.941***	894.252***	3,405.034***	61.944***	26.756***	283.862***
CEE Countries	11	11	11	11	11	11	11	11	11	11
Regions	61	61	61	61	61	61	61	61	61	61
Obs.	1,121	944	409	1,015	623	740	901	764	463	736

*Source*: Authors’ calculus.

*Note*: The symbol *** indicates significance at the 1% level.

### 4.2. Correlation analysis for both subsamples of regional subdivisions

As a second step in our analysis, we determined correlations between our predictors because we aimed at spotting potential multicollinearity problems that could bias our estimated results. According to standard practices, such multicollinearity issues usually become problematic when correlation coefficients exceed the 0.9 threshold. Such correlations were determined for both subsamples of regional subdivisions (see Tables 5 and 6).

**Table 5:** Correlation matrix for Western Europe regions.

Indicators	GDP	GVAD	ELET	DHI	IBA	LU	IRM	AP	HI	LFP
**GDP**	1									
**GVAD**	0.999	1								
**ELET**	−0.066	−0.06	1							
**DHI**	0.381*	0.379*	−0.555**	1						
**IBA**	0.107	0.107	−0.482*	0.370*	1					
**LU**	0.271*	0.269*	−0.508**	0.707**	0.387*	1				
**IRM**	0.758**	0.758**	−0.175	0.357*	0.296*	0.426*	1			
**AP**	0.255*	0.254*	0.142	0.123	−0.309*	−0.011	0.090	1		
**HI**	−0.124	−0.123	−0.14	−0.066	0.306*	0.230	0.180	−0.333	1	
**LFP**	0.956***	0.957***	0.072	0.209*	0.019	0.071	0.698**	0.245*	−0.161	1

*Source*: Authors’ calculus.

*Note*: The symbols ***, **, * indicate significance at the 1%, 5% and 10% levels.

**Table 6:** Correlation matrix for Central and Eastern Europe regions.

Indicators	GDP	GVAD	ELET	DHI	IBA	LU	IRM	AP	HI	LFP
**GDP**	1									
**GVAD**	0.999	1								
**ELET**	−0.579	−0.595	1							
**DHI**	0.693	0.698	−0.520**	1						
**IBA**	0.277	0.272	0.009	0.599**	1					
**LU**	0.841	0.843	−0.584**	0.755***	0.183	1				
**IRM**	0.034	0.012	0.218*	0.177	0.179	0.028	1			
**AP**	−0.119	−0.101	−0.243*	−0.122	−0.327*	−0.109	−0.257*	1		
**HI**	0.306	0.314	−0.309*	0.603**	0.489*	0.458*	−0.017	−0.347*	1	
**LFP**	0.322	0.329	−0.286*	−0.169	−0.077	−0.086	−0.444	0.034	−0.132	1

*Source*: Authors’ calculus.

*Note*: The symbols ***, **, * indicate significance at the 1%, 5% and 10% levels.

Table 5 displays the correlation coefficients for Western European regions. The highest significant correlation was established between the predictors labor utilization rate (LU) and disposable household income (DHI) (*r* = 0.71). The lowest correlation was established between the predictors titled labor force and participation (LFP) and disposable household income (DHI), that is *r* = 0.21.

Since none of our correlation coefficients exceeded the standard threshold of 0.9, we concluded that the risk of multicollinearity was low. Hence, this would not bias estimated results from the econometric modelling.

According to Table 6, in the case of Central and Eastern Europe regions, the highest significant correlation was established between the independent variables LU and DHI (*r* = 0.76). The lowest significant correlation was established between the predictors ELET and LFP (*r* = -0.29). Since the 0.9 standard threshold was not exceeded by any combination of predictors, we concluded that multicollinearity would not pose any problem for our results estimated via the proposed econometric models.

### 4.3. Econometric models

Table 7 presents the estimations of the first-difference GMM models testing the relationship between wellbeing-related infrastructure and economic growth.

**Table 7:** Econometric models corresponding to the dependent variables GDP and GVAD.

	VIF	Model 1 GDP	VIF	Model 2 GVAD	VIF	Model 3 GDP	VIF	Model 4 GVAD
**GDP(–1)**		0.202*** (8.393)	-	-	-	0.520*** (4.019)		-
**GVAD(–1)**		-	-	0.559*** (45.783)	-	-		0.360*** (2.616)
**ELET**	1.794	−0.002*** (−5.074)	1.792	0.0004*** (2.668)	3.017	0.007* (1.758)	3.017	0.007* (1.880)
**DHI**	3.195	0.406*** (23.977)	3.194	0.529*** (33.559)	7.805	0.178 (1.419)	7.805	0.103 (1.053)
**IBA**	1.867	−0.0001 (−0.987)	1.865	−0.001*** (−16.173)	3.481	0.001*** (3.011)	3.481	0.001 (1.494)
**LU**	3.111	0.008*** (8.604)	3.110	7.53 (0.100)	3.271	0.006 (0.910)	3.271	0.014 (1.560)
**IRM**	6.561	0.059*** (6.237)	6.559	−0.182*** (−34.269)	1.599	0.122 (1.323)	1.599	0.002 (0.017)
**AP**	1.348	0.004*** (6.482)	1.347	0.007*** (18.322)	1.747	0.012*** (3.089)	1.747	0.009** (2.179)
**HI**	1.678	0.113*** (11.018)	1.679	0.034*** (4.860)	2.012	0.152*** (2.844025)	2.012	0.114** (2.295)
**LFP**	5.292	0.735*** (16.538)	5.288	1.012*** (27.089)	1.833	−0.033 (−0.115)	1.832	−0.280 (−0.842)
**Cross-section effects**	-	Fixed	-	Fixed	-	Fixed	-	Fixed
**Period fixed**	-	Yes	-	Yes	-	Yes	-	Yes
**White period standard errors & covariance (d.f. corrected)**	-	Yes	-	Yes	-	Yes	-	Yes
**Hansen *J*-statistic**	-	138.520	-	73.635	-	12.162	-	14.356
**Prob(*J*-statistic)**	-	0.000	-	0.299	-	0.352	-	0.214
**AR(1)**	-	0.001	-	0.013	-	N/A	-	N/A
**AR(2)**	-	0.754	-	0.534	-	0.752	-	0.470
**Observations**	-	475	-	474	-	128	-	128
**Instrument rank**	-	80	-	77	-	20	-	20

*Source*: Authors’ calculus.

*Note*: Robust *t*-statistics are presented in parentheses. *, **, *** indicate statistical significance at the 10%, 5% and 1% levels. Multicollinearity was investigated by means of the variance inflation factor (VIF). For all econometric models, we concluded that multicollinearity did not pose a problem since all VIF values were below the standard threshold of 10. In addition, the White test rejected the null hypothesis of heteroscedasticity. The Arellano-Bond test investigated if errors were serially correlated. According to results, there was no first-order autocorrelation and second-order autocorrelation based on AR(1) and AR(2) tests. Furthermore, the validity of the GMM estimator was confirmed for AR(2): since the test was statistically insignificant, no second-order serial correlation was identified, hence it satisfied instrument validity. The Hansen *J*-statistic test of over-identifying restrictions was not significant, therefore the null hypothesis of valid instruments could not be rejected. This confirmed the validity of our econometric models.

The phenomenon of economic growth was proxied by the regional gross domestic product (GDP) and regional gross value added (GVAD). Model 1 and Model 2 estimated economic growth for Western European regions, while Model 3 and Model 4 estimated the phenomenon for Central and Eastern Europe regions. Under the name of each model, we indicated the proxy used for economic growth.

For **Model 1**, when examining the evolution of the regional gross domestic for the Western European regions, our results showed that most independent variables had a significant influence on GDP. Empirical results showed that the impact of ELET was negative. Hence, should this independent variable increase by one percent, GDP would decrease by 0.002 units. At the same time, should DHI, LU, IRM, AP, HI and LFP increase by one unit, GDP would rise by 0.41, 0.01, 0.06, 0.004, 0.11 and 0.74 units, respectively. Overall, the probabilities corresponding to the *J*-statistic test and the Arellano-Bond test for AR(2) indicated that the combined effect of the independent variables was statistically significant.

When considering **Model 2** and the regional gross value added as outcome, almost the same variables stayed relevant for regional economic growth. In this sense, when ELET, DHI, AP, HI and LFP augmented by one unit, GVAD would also increase by 0.0004, 0.53, 0.007, 0.034 and 1.01 units, respectively. Moreover, the factors IBA and IRM had a negative influence: should these factors improve by one unit, GVAD would decrease by 0.001 and 0.18, respectively. Overall, the probabilities corresponding to the *J*-statistic test and the Arellano-Bond test for AR(2) indicated that the combined effect of the independent variables was statistically significant.

We also examined the relationship between wellbeing-related infrastructure and economic growth proxies for the Central and Eastern Europe regions. According to **Model 3**, the independent variables ELET, IBA, AP and HI had a positive influence on economic growth: when these variables improved by one percent, regional GDP would also improve by 0.007, 0.001, 0.011 and 0.152 units, respectively. Hence, based on the probabilities corresponding to the *J*-statistic test and the Arellano-Bond test for AR(2), it can be stated that factors had a significant combined effect on economic growth proxied by regional GDP.

Regarding **Model 4**, empirical results showed that ELET, AP and HI remained relevant for the Central and Eastern European regions subsample. In this sense, GVAD would increase by 0.007, 0.009 and 0.114, respectively. All in all, the probabilities corresponding to the *J*-statistic test and the Arellano-Bond test for AR(2) indicated that the combined effect of the independent variables was statistically significant.

### 4.4. Multiplex network analysis

We ran a multiplex network analysis with the time series of all independent and dependent variables for both subsamples of European regions and estimated the relationship dynamics within each subsample through multiplex measurements. This type of analysis was used in Batrancea et al. [98, 99] and it is grounded on the following standard steps.

In the first place, the mathematical layout of a single-layer network should be presented. For this aim, the following elements are required:

- a set of agents, *R*_1_,…,*R*_*N*_ (i.e., European regional subdivisions);
- *N* nodes grouped in a set denoted by *V*, for mapping the network;
- the edge formation rule, namely ∀*i*, *j*, 1 ≤ *i*, *j* ≤ *N*, *i* ≠ *j*, (*R*_*i*_, *R*_*j*_) ∈ *E*.
- a time series of each region as *TS*_1_, *TS*_2_, …, *TS_N_*, for determining network interactions.

One should assign an edge between each pair of nodes because European regional subdivisions will interact to a certain degree. In addition, new layers are produced if the time series is modified. Moreover, the similarity of agents’ time series can serve as proxy for their interaction in an *N*-node network.

Network interactions are fundamental when determining network topology. We quantified the interaction degree by comparing time series that yielded distinct edges. Therefore, we chose the dynamic time warping (DTW) similarity measure, which is considered very effective for contrasting two time series while performing a nonlinear transformation to our data.

According to the theory, weighted graphs should be used to represent network architecture. Hence, the weighting function is denoted as *ω*: *E* → *ℝ* and defined on edges, while a weighted graph is represented by the triple *G* = (*V*,*E*,*ω*). In this context, the basic equation for the weighting function is: *ω*(*R*_*i*_, *R*_*j*_) ≡ *DTW* (*TS*_*i*_, *TS*_*j*_).

Complex systems can be represented by embedding edges into distinct layers. Nevertheless, one needs a variety of structural metrics to analyze a multiplex structure that emerges when similarity measures are applied to separate time series originating from each region. Each individual monoplex in the multiplex structure comprises DTW-weighted edges, while individual nodes stand for different regions. The value of the DTW variable defines the robustness of weighted edges based on the interaction strength and the strength of the node inside the multiplex topology. On the macroeconomic market, this would indicate the level of economic supremacy or the power of a nation. In order to determine the strength of a node in a layer, we sum up the edge weights that are next to that node.

Let us consider a system with *N* nodes and *M* weighted layers. Each layer can be associated with an adjacency matrix $A^{a}\;=\;[a_{ij}^{a}]$, where *α* denotes the layer index. As stated before, we formed networks by using DTW of multivariate time series of each region. All networks in the multiplex layers were weighted and undirected. We consider the strength of a node in an *α* layer as the total of edge weights adjacent to that node. We denote the strength of a node *i* in *α* layer with $s_{i}^{\alpha }$. Moreover, the weighted edge overlapped degree o_*i*_ of a node *i* can be defined as the total strength with $o_{i\;}=\;\sum_{1\leq \alpha \leq M}s_{i}^{\alpha }.$

In the case of multiplex networks, one must investigate how the strength of a node is distributed between layers. Hence, we computed the aggregated topological strength *s*_*i*_ and the strength of the nodes in each layer $s_{j}^{\alpha }$, ordering the nodes according to their aggregated topological strength, by using:

α∈{GDP,GVAD,DHI,HI,LU,LFP,AP,ELET,IBA,IRM}.

We computed the Kendall rank correlation coefficient, τ_*s*_, which evaluates the similarity of two ranked sequences of data *X* and *Y* to determine correlations of node strengths. Because it makes no assumptions about distributions and has values in the range of [–1,1], this correlation coefficient τ_*s*_ is a nonparametric measure of statistically dependency between two ranks. If rankings are identical, τ_*s*_ (*X*,*Y*) =1 If one rank is precisely the opposite of the other, τ_*s*_ (*X*, *Y*) =-1. Moreover, if variables *X* and *Y* are independent, τ_*s*_ (*X*,*Y*) =0.

**Fig 1:**
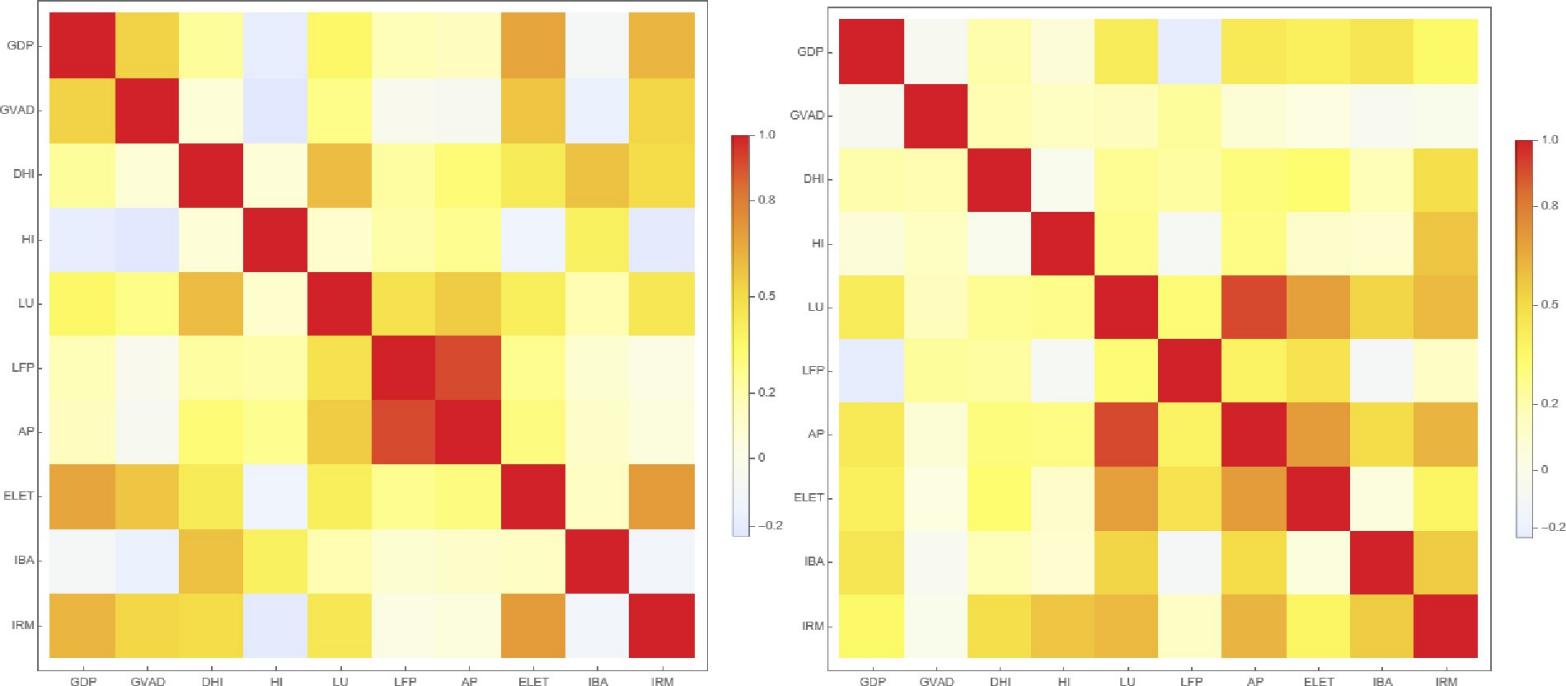
The Kendall correlation coefficient for Central and Eastern Europe regions (left hand side) and Western Europe regions (right hand side).

Fig 1 displays the values of τ_*s*_ obtained for the rankings of each pair of variables, as a heat map.

When considering the interaction network of European regions, network clusters with close relationships become evident. Therefore, the impact of any economic behavior on a particular system may be explained through variables that make up network links. The earliest indicators of varied density can be seen in the clusters formed by the Kendall correlation coefficient. When it comes to interactions, the effect of regions grows with every subsequent multiplex tier. From an economic standpoint, this approach reveals whether a region dominates the interaction or whether it shares cooperation-related traits with other regions.

It is worth noting that, by observing bright yellow sections of the heat map, a node’s strength in the IBA layer is unrelated to its strength in most of the other layers for the CEE regions. In addition, the GVAD layer is unrelated to its strength in most of the other layers for the WE regions.

The multiplex participation coefficient *P*(*i*) of node *i* determines the weighted contribution of that node to network communities and it can be defined as follows:

$$P(i)=\frac{M}{M-1}\left(1-\sum_{\alpha =1}^{M}{\left(\frac{s_{i}^{\alpha }}{o_{i}}\right)}^{2}\right).$$

Hence, *P*(*i*) shows whether the links of node *i* are evenly dispersed across *M* levels or concentrated in one/few layers. The bigger the value of *P*(*i*), the more evenly dispersed are node links in the *M* layers of the multiplex. The average of *P*(*i*) over all nodes represents the participation coefficient *P* of the entire multiplex.

**Fig 2:**
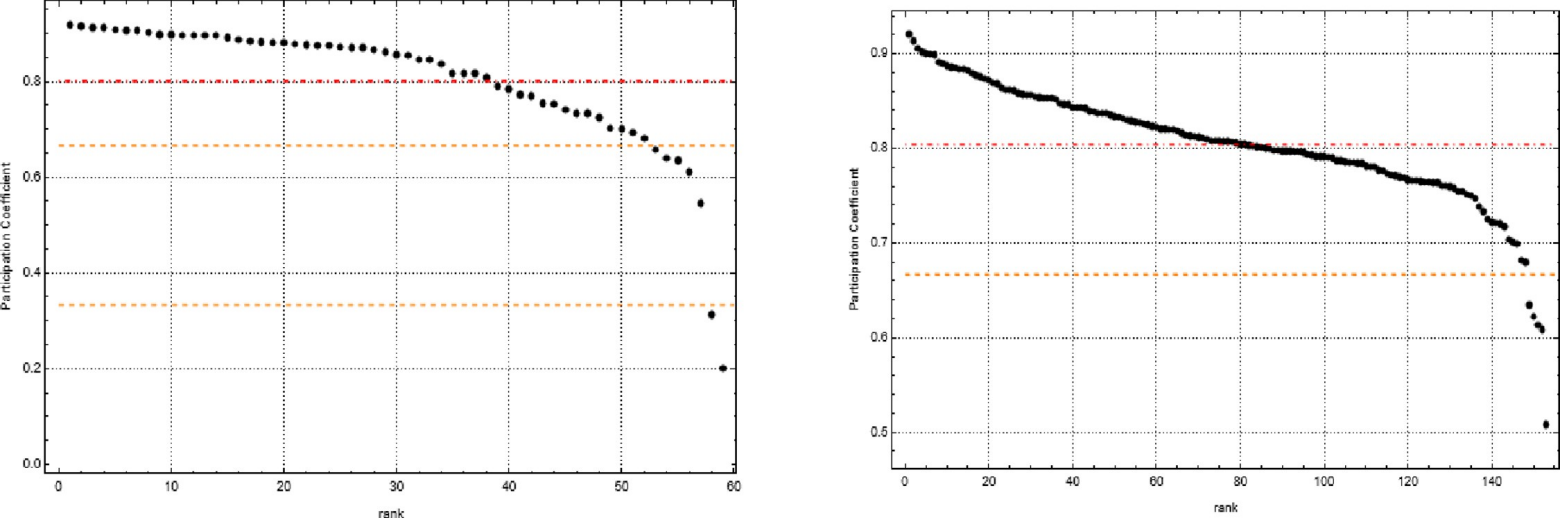
Rank distribution of participation coefficients for Central and Eastern Europe regions (left hand side) and the Western Europe regions (right hand side). *Note*: Orange dashed lines represent focuses, red dot-dashed lines represent average participation coefficients.

Fig 2 displays the distributions of *P*(*i*) for the multilayer network. Although the multiplex average participation coefficient is 0.8, we identified a distribution of *P*(*i*) in the range [0,1]. This variation indicates that the network includes different amounts of node engagement in each of its ten layers. Next, we classified multiplex nodes by analyzing their *P*(*i*) and overlapping strength because the strength represents the overall importance of the node regarding the number of incident edges. *P*(*i*) gives information about the distribution of incident edges across layers. We distinguished three types of nodes: *focused*, with $0\;\text{<}\;P(i)\;\text{<}\frac{1}{3}$; *mixed*, with $\frac{1}{3}\leq P(i)\;\text{<}\frac{1}{2}$; *truly multiplex*, with $P(i)\;\geq \frac{2}{3}$.

The *P*(*i*) rank distribution shows that averages of both CEE and WE regions were almost identical, with most coefficients being above $\frac{2}{3}$. We concluded that analyzing both types of regions through multiplexes was meaningful. Moreover, none of the WE regions became focused.

**Fig 3:**
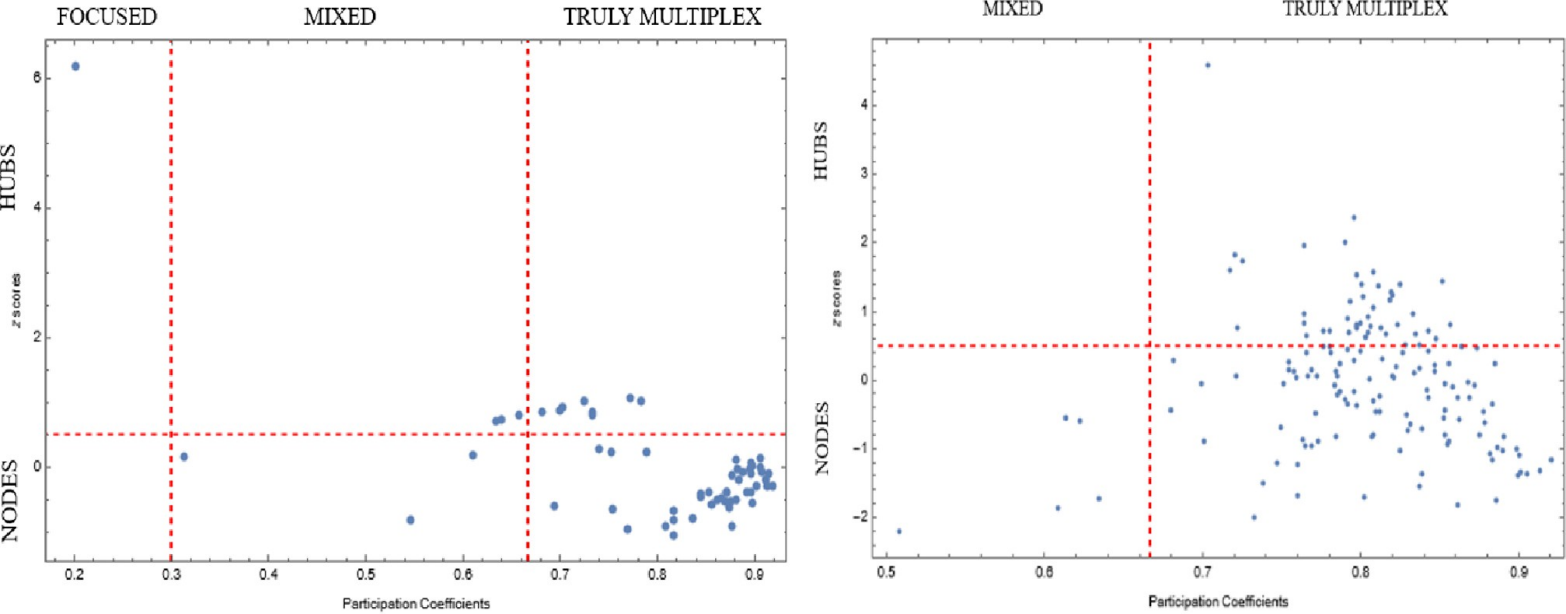
Plotting the multiplex participation coefficient *P*(*i*) with the *Z*-score of total overlapping strength yields a map of the responsibilities of nodes in a multilayer network for Central and Eastern Europe regions (left hand side) and Western Europe regions (right hand side). *Note*: Even if two nodes have the same *Z*-score value, their functions might change significantly depending on the value of their multiplex participation coefficient.

Instead of the overlapping strength, we used the corresponding *Z*-score strength defined as:

$$z(o_{i})=\;\frac{o_{i}-\mu_{o}}{\sigma_{o}},$$

where μ_*o*_ denotes the mean and σ_*o*_ denotes the standard deviation of the overlapping strengths. We identified hubs (with *z*(*o*_*i*_) ≥ 0.5) from ordinary nodes (with *z*(*o*_*i*_) < 0.5) based on the *Z*-score of their overlapping strength. Consequently, we could define six types of nodes by considering the *P*(*i*) of a node and its total overlapping strength *o*_*i*_, as shown in Fig 3, where each node is represented as a point in the (*P_i_*, *z*(*o*_*i*_)) plane.

The *P*(*i*) values and *Z*-scores are presented in Table 8 (CEE regions) and Table 9 (WE regions).

**Table 8:** Multiplex participation coefficient *P*(*i*) with *Z*-scores (CEE regions).

Region	*P*(*i*)	*z*(*o*_*i*_)	Region	*P*(*i*)	*z*(*o*_*i*_)	Region	*P*(*i*)	*z*(*o*_*i*_)
Prague	0.8972	−0.5467	Lesser Poland	0.7402	0.2877	East Slovakia	0.8833	−0.1965
Central Bohemian Region	0.9067	−0.0131	Silesia	0.6108	0.1723	Eastern Slovenia	0.9179	−0.3029
Southwest	0.9148	−0.0932	Greater Poland	0.2007	6.1886	Western Slovenia	0.8962	−0.3808
Northwest	0.8952	−0.0318	West Pomerania	0.7837	1.0073	North West (Bulgaria)	0.8172	−1.0549
Northeast	0.9070	−0.0784	Lubusz	0.7336	0.7970	North Central (Bulgaria)	0.7691	−0.9651
Southeast	0.8810	0.0978	Lower Silesia	0.7526	0.2336	North East (Bulgaria)	0.8077	−0.9032
Central Moravia	0.9059	0.1341	Opole Region	0.7895	0.2375	South East (Bulgaria)	0.8162	−0.8288
Moravia Silesia	0.8962	0.0683	Kuyavian Pomerania	0.7724	1.0648	South West (Bulgaria)	0.8169	−0.6791
Estonia	0.9121	−0.1907	Warmian Masuria	0.7326	0.8568	South Central (Bulgaria)	0.8357	−0.8039
Budapest	0.7536	−0.6494	Pomerania	0.6999	0.8802	Adriatic Croatia	0.6936	−0.6077
Pest	0.8447	−0.4628	Lodzkie	0.6392	0.7332	Continental Croatia	0.5459	−0.8206
Central Transdanubia	0.8770	−0.1347	Swietokrzyskie	0.6578	0.7993	North West (Romania)	0.8703	−0.5440
Western Transdanubia	0.8827	−0.0383	Lublin Province	0.7242	1.0153	Center (Romania)	0.8662	−0.4926
Southern Transdanubia	0.8451	−0.4235	Podkarpacia	0.7021	0.9119	North East (Romania)	0.8552	−0.5835
Northern Hungary	0.8920	−0.3910	Podlaskie	0.6344	0.7166	South East (Romania)	0.8620	−0.5064
Northern Great Plain	0.9124	−0.2869	Warsaw	0.3133	0.1669	South Muntenia (Romania)	0.8741	−0.6276
Southern Great Plain	0.8957	−0.0986	Mazowiecki Region	0.6817	0.8414	Bucharest Ilfov (Romania)	0.8771	−0.9184
Latvia	0.9022	−0.2843	Bratislava Region	0.8714	−0.3869	South West Oltenia (Romania)	0.8812	−0.5092
Vilnius Region	0.8747	−0.5288	West Slovakia	0.8979	0.0205	West (Romania)	0.8704	−0.3967
Central and Western Lithuania	0.8534	−0.3935	Central Slovakia	0.8875	−0.0763			

*Source*: Authors’ calculus.

**Table 9:** Multiplex participation coefficient *P*(*i*) with *Z*-scores (WE regions).

Region	*P*(*i*)	*z*(*o*_*i*_)	Region	*P*(*i*)	*z*(*o*_*i*_)	Region	*P*(*i*)	*z*(*o*_*i*_)
Burgenland	0.7846	−0.8208	Saarland	0.7995	0.8396	South Holland	0.8158	0.6819
Lower Austria	0.8721	−0.0816	Saxony	0.7928	0.7044	Zeeland	0.7512	−0.0515
Vienna	0.8772	−0.4648	Saxony Anhalt	0.8045	0.9289	North Brabant	0.7693	0.1525
Carinthia	0.8119	−0.2416	Schleswig Holstein	0.8231	0.8162	Limburg	0.7763	0.4909
Styria	0.8559	0.2424	Thuringia	0.8082	1.0583	North (Portugal)	0.8848	0.2414
Upper Austria	0.8528	−0.0542	Attica	0.8995	−1.3809	Algarve	0.7915	0.4378
Salzburg	0.8429	−0.2461	North Aegean	0.8368	−1.5409	Central Portugal	0.8734	0.4618
Tyrol	0.7973	−0.3655	South Aegean	0.8555	−0.8825	Metropolitan Area of Lisbon	0.8328	0.9713
Vorarlberg	0.7913	−0.3477	Crete	0.9202	−1.1546	Alentejo	0.8428	0.4177
Brussels Capital Region	0.8109	1.3768	Eastern Macedonia Thrace	0.9133	−1.3191	Autonomous Region of the Azores	0.7496	−0.6898
Flemish Region	0.7802	0.4948	Central Macedonia	0.9001	−1.0991	Autonomous Region of Madeira	0.7633	−0.8605
Wallonia	0.8008	1.3928	Western Macedonia	0.8854	−1.7572	Galicia	0.8578	−0.0961
Copenhagen Region	0.7932	1.1411	Epirus	0.9053	−1.3791	Asturias	0.7719	0.0596
Zealand	0.8464	0.2247	Thessaly	0.8895	−1.0209	Cantabria	0.7837	−0.0861
Southern Denmark	0.8637	0.4866	Ionian Islands	0.8612	−1.8302	Basque Country	0.833494	0.1100
Central Jutland	0.8566	0.7992	Western Greece	0.8831	−1.1669	Navarra	0.8206	0.0352
Northern Jutland	0.8371	0.1796	Central Greece	0.8819	−1.0733	LaRioja	0.7216	0.7696
Western Finland	0.8193	1.2793	Peloponnese	0.8751	−0.8104	Aragon	0.8064	0.7840
Helsinki Uusimaa	0.8429	0.7161	Northern and Western (Ireland)	0.7602	−1.2402	Madrid	0.7842	0.1228
Southern Finland	0.8474	0.5946	Southern And Eastern (Ireland IE05)	0.7013	−0.8869	Castile And León	0.8057	0.0063
Eastern and Northern Finland	0.8681	−0.0258	Southern and Eastern (Ireland IE06)	0.6794	−0.4362	Castile LaMancha	0.7976	0.7812
Åland	0.6132	−0.5559	Piedmont	0.8387	−0.7099	Extremadura	0.8907	−0.8275
Île de France	0.7651	−0.9642	Aosta Valley	0.7384	−1.5148	Catalonia	0.7542	0.2697
Centre Val de Loire	0.7974	0.7642	Liguria	0.8619	−0.5743	Valencia	0.8613	−0.2483
Bourgogne-Franche-Comté	0.7759	0.7168	Lombardy	0.8021	−1.7076	Balearic Islands	0.8687	−0.2676
Normandy	0.7643	0.8235	Abruzzo	0.7954	−0.1572	Andalusia	0.8287	−0.5017
Hauts de France	0.8278	0.5152	Molise	0.7468	−1.2032	Murcia	0.8833	−0.3581
Grand Est	0.7913	0.9082	Campania	0.7851	0.0651	Ceuta	0.6346	−1.7300
Pays de la Loire	0.7658	0.6587	Apulia	0.8296	−0.7347	Melilla	0.7329	−1.9972
Brittany	0.7803	0.7079	Basilicata	0.8075	−0.8115	Canary Islands	0.8787	−0.6197
Nouvelle Aquitaine	0.8203	1.2381	Calabria	0.8868	−0.9724	Stockholm	0.8313	−0.6423
Occitanie	0.8514	1.4378	Sicily	0.8532	−0.79667	East Middle Sweden	0.8266	0.4092
Auvergne Rhône Alpes	0.8044	0.6849	Sardinia	0.8094	−0.4722	Småland with Islands	0.8001	0.4307
Provence Alpes Côte d’Azur	0.7977	0.8189	Province Bolzano Bozen	0.9013	−1.3519	South Sweden	0.8368	0.5172
Corsica	0.7901	−0.2746	Province Trento	0.8546	−0.9454	West Sweden	0.8345	0.6670
Guadeloupe	0.8082	−0.3094	Veneto	0.8117	−0.4544	North Middle Sweden	0.8135	0.3184
Martinique	0.7869	−0.1766	Friuli Venezia Giulia	0.8536	−0.4304	Central Norrland	0.7667	0.0646
French Guiana	0.7602	−1.6817	Emilia Romagna	0.8986	−1.0026	Upper Norrland	0.7869	0.2450
La Réunion	0.8419	−0.1464	Tuscany	0.8527	−0.5606	North East England	0.7973	1.5341
Mayotte	0.5084	−2.2147	Umbria	0.8069	−0.8153	North West England	0.7249	1.7468
Baden Württemberg	0.7729	−0.8925	Marche	0.7848	−0.2163	Yorkshire and the Humber	0.7903	1.9995
Bavaria	0.7686	−0.9577	Lazio	0.8246	−1.0245	East Midlands	0.7637	1.9665
Berlin	0.7204	1.83747	Luxembourg	0.8390	−1.3603	West Midlands	0.7961	2.3669
Brandenburg	0.8012	1.2063	Groningen	0.7539	0.1566	East England	0.7644	0.9640
Bremen	0.7173	1.6046	Friesland	0.7579	0.1338	Greater London	0.6223	−0.5907
Hamburg	0.8198	0.0696	Drenthe	0.7211	0.0564	South East England	0.6817	0.2832
Hesse	0.7713	−0.4940	Overijssel	0.7812	0.4065	South West England	0.8251	1.4009
Mecklenburg Vorpommern	0.8180	1.1707	Gelderland	0.7961	0.2890	Wales	0.8129	0.7739
Lower Saxony	0.7660	0.3909	Flevoland	0.7589	0.04085	Scotland	0.8076	1.5879
North Rhine Westphalia	0.6989	−0.0636	Utrecht	0.8464	0.1335	Northern Ireland	0.7035	4.602
Rhineland Palatinate	0.8029	0.6258	North Holland	0.8226	0.1921	Malta	0.6086	−1.8582

*Source*: Authors’ calculus.

According to our estimations, the time series rank correlations can be interpreted as follows. The percentage of homes having internet broadband access (IBA) is the weakest variable for determining the similarity connections for Central and Eastern Europe regions. There is, nevertheless, a significant correlation between the variables capturing internet broadband access and disposable household income. In the case of WE regions, this link is irrelevant. Regional GDP and regional GVAD have a strong correlation for CEE regions, but a lower correlation for WE regions. Air pollution was highly correlated with labor force and participation in the case of CEE regions, while it was also highly correlated with labor utilization rate for the WE regions.

According to Tables 8 and 9, the truly multiplex hubs for the CEE regions were: West Pomerania, Lubusz, Kuyavian Pomerania, Warmian Masuria, Pomerania, Swietokrzyskie, Lublin Province, Podkarpacia and Mazowiecki. Moreover, the mixed hubs were Lodzkie and Podlaskie. Even though Greater Poland emerged as a hub region, its participation coefficient makes it to be considered an outlier. Since most nodes in the WE regions were truly multiplex, we mainly considered them hubs. Overall, hubs were noticed in regions from the UK, Germany and France. Moreover, mostly Nordic countries tended to be hubs.

**Fig 4:**
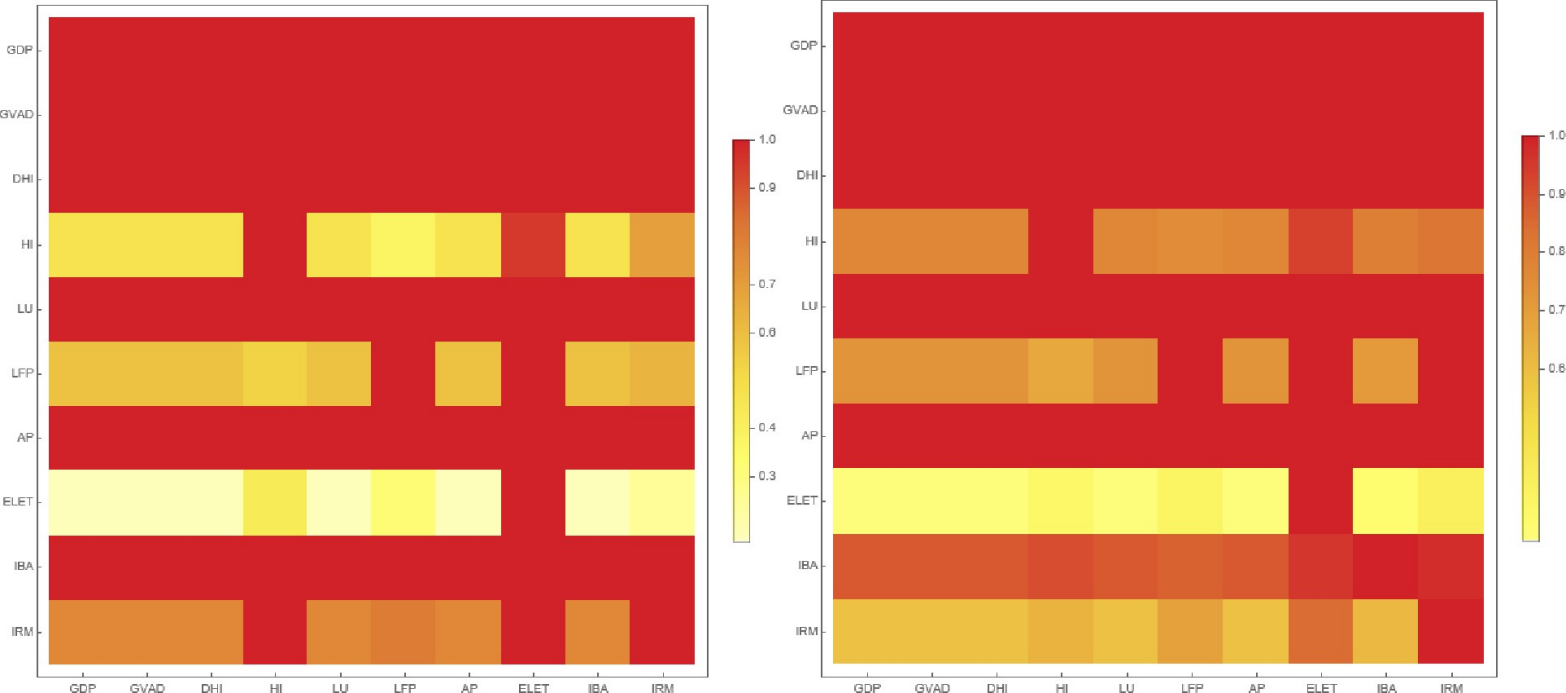
Conditional probabilities of links within each layer for Central and Eastern Europe regions (left hand side) and Western Europe regions (right hand side).

We also aimed to quantify the relevance of each layer after proposing certain measurements for the role of individual nodes in a multiplex. The conditional probability of detecting a connection at layer *α* (given an edge connecting the identical nodes at layer *α*’ exists) is:

$$\mathbb{P}(a_{ij}|a{'}_{ij})=\frac{\sum_{ij}a_{ij}a{'}_{ij}}{\sum_{ij}a_{ij}},$$

where the denominator is equal to the number of edges at layer *α*. Fig 4 shows the conditional probability in a heat map.

As Fig 4 shows, the first row of the heat map indicates the probability of edges of the GDP layer to be present in other layers. When looking at the overall matrix, every edge from the GDP, GVAD, LU, DHI, AP and IBA layers may be found in other layers. Hence, it can be stated that these were the most effective factors. The ELET variable had the lowest efficiency for CEE regions, but it is more efficient for WE regions. The dominance of the IBA layer manifested in the CEE regions subsample weakened for WE regions.

**Fig 5:**
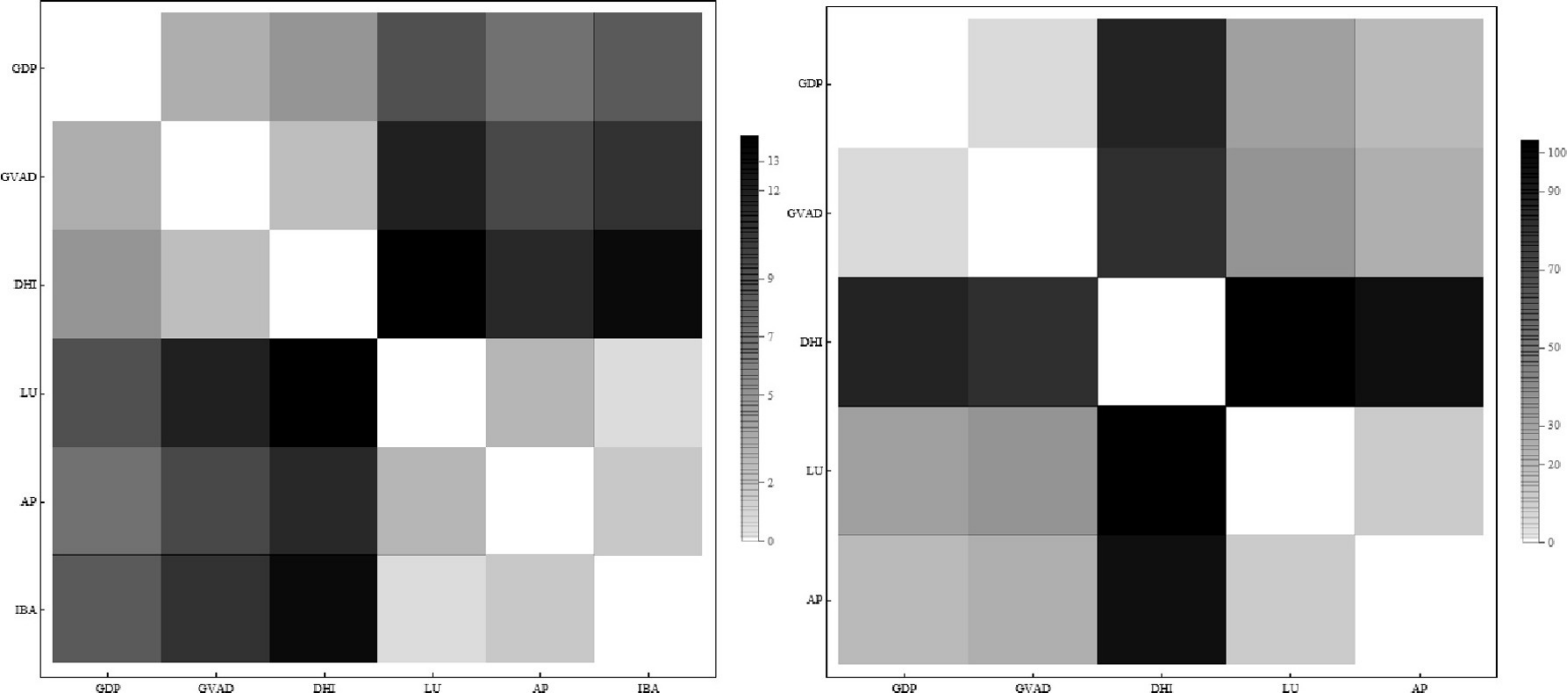
Wasserstein-1 distance matrix of dominant layers for Central and Eastern Europe regions (left hand side) and Western Europe regions (right hand side).

Edges and their connection strength become important when considering the weight of multiplexes employed in our study. Hence, we compared the Wasserstein-1 distances regarding node strength distributions in each dominant layer to identify those with the greatest node strength similarity. Fig 5 presents the comparison results.

Based on Fig 5, it can be stated that the least similar distributions emerged through the DHI and LU layers for CEE regions. Hence, the variables GDP, GVAD, AP and IBA were useful in forming multi-regional correlations. In terms of the Wasserstein-1 distance, a clustering was noticed among the prominent variables. For that matter, GDP, GVAD, DHI (on one side) and LU, AP and IBA (on the other side) clustered individually. For WE regions, DHI emerged as the most dominant layer. Moreover, a clustering behavior was noticed for GDP, GVAD, LU and AP.

## 5. Discussion and concluding remarks

The present study reports on an empirical investigation testing the impact of wellbeing-related infrastructure on regional economic growth with NUTS 2 data from 151 Western Europe regional subdivisions and 61 Central and Eastern Europe regional subdivisions. Bearing in mind that economic growth is a major phenomenon for the economy of any country and region, it should be examined in the long run. Hence, we chose to analyze the relationship for the decades 2001–2020.

We conducted a panel data analysis via the first-difference generalized method of moments (GMM) approach and cross-section fixed effects. Starting from the extant scientific literature, we selected ten variables of interest to conduct our investigation, which were retrieved from the OECD regional database. Economic growth was proxied by regional GDP and regional gross value added. The set of predictors that accounted for wellbeing-related infrastructure included the following variables: air pollution, housing indicator, internet broadband access, disposable household income, inter-regional mobility, labor force and participation, labor utilization rate and rate of early leavers from education and training. In our view, these predictors capture essential elements that drive people’s wellbeing state, considering that wellbeing depends heavily on the access to basic resources [100, 101].

Our focus on wellbeing-related infrastructure (i.e., facilities and capacities of accessing facilities to achieve wellbeing) was motivated by the fact that considerations on citizens’ wellbeing lie at the core of the European Union principles. Furthermore, the EU has also advanced the idea that economic growth and wellbeing are organically intertwined.

Empirical results showed that the chosen indicators played a fundamental role for the economies of these regions, thus supporting the two research hypotheses. Our findings suggest that, in the case of *Western Europe regions*, the strongest significant impact was triggered by the predictors disposable housing income, inter-regional mobility, housing indicator, labor force and participation. These results are sensible and according to expectations: 1) WE regions generally register higher GDP per capita and, consequently, people gain more money to allocate for household-related expenditure; 2) labor force mobility is more intense and rather common in these regions, which have higher percentages of renters on the housing market; 3) housing facilities are important for the overall wellbeing, although the rate of owned households is lower than in CEE regions; 4) having developed economies, WE regions present more job opportunities, attracting higher percentages of the active population. The variable air pollution had a low but significant impact on economic performance. Although increased pollution may translate into more economic activity, in the long run the variable affects citizens’ wellbeing because it puts a toll on the overall health state and work capacities.

At the same time, we found that almost all variables had a positive influence on economic growth (with few exceptions). The negative impact of early leavers from education and training is as expected: the more skilled and educated potential employees are, the more businesses thrive and economies grow.

For the subsample of *Central and Eastern Europe regions*, all significant variables had a positive impact on both economic growth proxies. The most notable variables were the housing indicator, air pollution and internet broadband access. The housing indicator had the strongest impact on economic progress on the account that CEE regions have the highest rate of property ownership in the world [102], with Romania, Slovakia, Croatia, Lithuania, Hungary and Poland ranking among the first 10 countries. Home ownership is regarded as fundamental in CEE countries and an important element of the wellbeing state. The influence of internet access was low but significant, in line with Goldbeck and Lindlacher [103]. In today’s digitalized economies, internet broadband access (i.e., internet access at download speeds of minimum 256 kbit/s) is crucial because it facilitates business, education, everyday life activities, entertainment, trade, job opportunities. This significant impact was expected considering that CEE regions have been hosting some of the largest IT hubs in Europe and benefit from very fast internet connections. Counterintuitively, early leavers from education and training had a weak but positive influence. Generally, regions should aim for mitigating the percentage of citizens aged 18–24 who exit education and training programs, because a poor formal development affects the set of skills requested on the labor market (i.e., people are harder to employ). Yet, a possible explanation could be that lower-educated people may increase economic growth of CEE regions by activating in sectors not requiring highly trained workers (e.g., agriculture, constructions, manufacturing, wholesale and retail trade, transportation and storage).

With some exceptions, our predictors had a relevant influence on both CEE and WE subsamples. Notable differences stemmed from the strength of the influence and the sign of the relationships between economic growth and designated predictors. In the case of CEE regions, economic growth was not influenced by disposable household income, labor utilization rate, inter-regional mobility or labor force and participation.

Aside from the econometric modelling of economic growth, we conducted a multiplex network analysis on the regional data, which prompted important results on regional networks. Following the standard multiplex procedure, the topological layer analysis indicated that regions were mostly hub nodes in our multiplex models. This result indicated that our multiplex approach for analyzing variables and subregional dependencies was effective. For both CEE and WE regions, most nodes were truly multiplex, namely hubs or subregions highly interconnected with the overall regional economy through the chosen variables. In the case of CEE regions, the following regional subdivisions were truly multiplex hubs: West Pomerania, Lubusz, Kuyavian Pomerania, Warmian Masuria, Pomerania, Swietokrzyskie, Lublin Province, Podkarpacia and Mazowiecki. In addition, Lodzkie and Podlaskie were mixed hubs. Even though Greater Poland emerged as a hub region (due to its participation coefficient), we considered it as an outlier. Since most nodes in WE regions were truly multiplex, we considered them hubs. In this context, most WE hubs were registered in countries with strong economies such as France, Germany, the UK, the Nordic countries.

One of the most important outcomes regarding the multiplex analysis is the occurrence of connections among variables. The similarity of subregions in terms of variables shows the effectiveness of that variable. Hence, the most influential variables were GDP, gross value added, labor utilization rate, disposable household income, air pollution and internet broadband access for both CEE and WE subsamples. We also observed that, in the case of CEE regions, early leavers from education and training had a much lower impact than for WE regions. To determine the most important variable, we studied the similarity of distributions of possibilities with the Wasserstein-1 distance. According to results, disposable household income and labor utilization emerged as weaker variables for the CEE regions, while disposable income was the dominant variable for WE regions. We also identified a clustering behavior for the variables GDP, gross value added, air pollution and labor utilization.

As with any scientific study, our analysis is prone to certain limitations. First, the category of wellbeing-related infrastructure comprises eight variables related to the environment, housing, internet facilities, financial resources, labor force mobility and skills. Upcoming studies might consider broadening the set of predictors with other variables concerning life satisfaction, work-life balance, safety or civic engagement. Second, the period of analysis spanned two decades, after the beginning of the 21^st^ century. Future studies might consider expanding the time frame to examine regional economic growth across several decades while scrutinizing the impact of different global crises. Third, the study is focused on regional subdivisions from countries in Western Europe and Central and Eastern Europe, which belonged to EU-28. Other investigations could test the relationship on NUTS 2 and even NUTS 3-type data for aspiring members of the European Union.

All in all, our study brings important insights on policies that could assist WE and CEE regions in growing their economic performance. On the one hand, WE regions would benefit a great deal if companies were incentivized to create more job opportunities, since this way labor force and participation on the job market would increase, yielding higher income resources for households and more products/services available to the general public. On the other hand, CEE regions would progress more via improving housing facilities, intensifying the digitalization of public and private sectors and via intense e-commerce activities [104].

## Supporting information

S1 Appendix (DOCX)
