# Supplementary material for: Empirical investigation on how wellbeing-related infrastructure shapes economic growth: Evidence from the European Union regions
Source: PLoS One. 2023 Apr 19;18(4):e0283277. doi: 10.1371/journal.pone.0283277 (PMC10115347; doi:10.1371/journal.pone.0283277)
Supplement: S1 Appendix — (DOCX) [file pone.0283277.s001.docx]

The 212 NUTS 2 regions used in this study are presented below.

**Central and Eastern Europe regions:**

**Bulgaria**

1. North West
2. North Central
3. North East
4. South East
5. South West
6. South Central

**Czech Republic**

1. Prague
2. Central Bohemian Region
3. Southwest
4. Northwest
5. Northeast
6. Southeast
7. Central Moravia
8. Moravia Silesia

**Estonia**

1. Estonia

**Croatia**

1. Adriatic Croatia
2. Continental Croatia

**Latvia**

1. Latvia

**Lithuania**

1. Vilvius Region
2. Central and Western Lithuania

**Hungary**

1. Budapest
2. Pest
3. Central Transdanubia
4. Western Transdanubia
5. Southern Transdanubia
6. Northern Hungary
7. Northern Great Plain
8. Southern Great Plain

**Poland**

1. Lesser Poland
2. Silesia
3. Greater Poland
4. West Pomerania
5. Lubusz
6. Lower Silesia
7. Opole Region
8. Kuyavian Pomerania
9. Warmian Masuria
10. Pomerania
11. Lodzkie
12. Swietokrzyskie
13. Lublin Province
14. Podkarpacia
15. Podlaskie
16. Warsaw
17. Mazowiecki Region

**Romania**

1. North-West
2. Center
3. North-East
4. South East
5. South Muntenia
6. Bucharest Ilfov
7. South West Oltenia
8. West

**Slovenia**

1. Eastern Slovenia
2. Western Slovenia

**Slovakia**

1. Bratislava Region
2. East Slovakia
3. Central Slovakia
4. West Slovakia

**Western Europe regions:**

**Austria**

1. Burgenland
2. Lower Austria
3. Vienna
4. Carinthia
5. Styria
6. Upper Austria
7. Salzburg
8. Tyrol
9. Vorarlberg

**Belgium**

1. Brussels Capital Region
2. Flemish Region
3. Wallonia

**Denmark**

1. Copenhagen Region
2. Zealand
3. Southern Denmark
4. Central Jutland
5. Northern Jutland

**Finland**

1. Western Finland
2. Helsinki-Uusimaa
3. Southern Finland
4. Eastern and Northern Finland
5. Åland

**France**

1. Île-de-France
2. Centre - Val de Loire
3. Bourgogne-Franche-Comté
4. Normandy
5. Hauts-de-France
6. Grand Est
7. Pays de la Loire
8. Brittany
9. Nouvelle-Aquitaine
10. Occitanie
11. Auvergne-Rhône-Alpes
12. Provence-Alpes-Côte d’Azur
13. Corsica
14. Guadeloupe
15. Martinique
16. French Guiana
17. La Réunion
18. Mayotte

**Germany**

1. Baden-Württemberg
2. Bavaria
3. Berlin
4. Brandenburg
5. Bremen
6. Hamburg
7. Hesse
8. Mecklenburg-Vorpommern
9. Lower Saxony
10. North Rhine-Westphalia
11. Saarland
12. Saxony
13. Saxony-Anhalt
14. Schleswig-Holstein
15. Thuringia

**Greece**

1. Attica
2. North Aegean
3. South Aegean
4. Crete
5. Eastern Macedonia, Thrace
6. Central Macedonia
7. Western Macedonia
8. Epirus
9. Thessaly
10. Ionian Islands
11. Western Greece
12. Central Greece
13. Peloponnese

**Ireland**

1. Northern and Western (IE)
2. Southern and Eastern (IE05)
3. Southern and Eastern (IE06)

**Italy**

1. Piedmont
2. Aosta Valley
3. Liguria
4. Lombardy
5. Abruzzo
6. Molise
7. Campania
8. Apulia
9. Basilicata
10. Calabria
11. Sicily
12. Sardinia
13. Province of Bolzano-Bozen
14. Province of Trento
15. Veneto
16. Friuli-Venezia Giulia
17. Emilia-Romagna
18. Tuscany
19. Umbria
20. Marche
21. Lazio

**Luxemburg**

1. Luxemburg

**Malta**

1. Malta

**The Netherlands**

1. Groningen
2. Friesland
3. Drenthe
4. Overijssel
5. Gelderland
6. Flevoland
7. Utrecht
8. North Holland
9. South Holland
10. Zeeland
11. North Brabant
12. Limburg

**Portugal**

1. North
2. Algarve
3. Central Portugal
4. Metropolitan area of Lisbon
5. Alentejo
6. Autonomous Region of the Azores
7. Autonomous Region of Madeira

**Spain**

1. Galicia
2. Asturias
3. Cantabria
4. Basque Country
5. Navarra
6. La Rioja
7. Aragon
8. Madrid
9. Castile and León
10. Castile-La Mancha
11. Extremadura
12. Catalonia
13. Valencia
14. Balearic Islands
15. Andalusia
16. Murcia
17. Ceuta
18. Melilla
19. Canary Islands

**Sweden**

1. Stockholm
2. East Middle Sweden
3. Småland with Islands
4. South Sweden
5. West Sweden
6. North Middle Sweden
7. Central Norrland
8. Upper Norrland

**United Kingdom**

1. North East England
2. North West England
3. Yorkshire and The Humber
4. East Midlands
5. West Midlands
6. East of England
7. Greater London
8. South East England
9. Wales
10. Scotland
11. Northern Ireland
